# Transcriptomics and Proteomics of *Haemonchus contortus* in Response to Ivermectin Treatment

**DOI:** 10.3390/ani13050919

**Published:** 2023-03-03

**Authors:** Yang Liu, Xiaomin Wang, Xiaoping Luo, Rui Wang, Bintao Zhai, Penglong Wang, Junyan Li, Xiaoye Yang

**Affiliations:** 1 School of Life Sciences, Ningxia University, Yinchuan 750021, China; 2 College of Veterinary Medicine, Inner Mongolia Agricultural University, Hohhot 010018, China; 3 The Bureau of Agriculture and Animal Husbandry of Kalaqin Banner, Chifeng 024400, China; 4 Inner Mongolia Academy of Agriculture and Animal Husbandry Sciences, Hohhot 010030, China; 5 Key Laboratory of Veterinary Pharmaceutical Development, Lanzhou Institute of Husbandry and Pharmaceutical Sciences, Chinese Academy of Agricultural Sciences, Ministry of Agriculture, Lanzhou 730050, China; 6 Department of Veterinary Parasitology, College of Veterinary Medicine, China Agricultural University, Beijing 100193, China

**Keywords:** ivermectin, *Haemonchus contortus*, transcriptomics, proteomics

## Abstract

**Simple Summary:**

*Haemonchus contortus* has a serious impact on the gastrointestinal health of ruminants and the economic sustainable development of animal husbandry. In this study, we examined transcriptomic and proteomic differences in ivermectin-resistant and -susceptible strains of *H. contortus* before and after Ivermectin (IVM) treatment. The results of two omics association analyses showed that UDP-glycosyltransferases (*UGT*), glutathione S-transferase (*GST*), cytochrome P450 (*CYP*), and p-glycoprotein (*Pgp*) genes play important roles in *H. contortus* drug resistance. Our study may provide useful data and new targets for research on the resistance response of *H. contortus.*

**Abstract:**

A major problem faced by the agricultural industry is the resistance of *Haemonchus contortus* to anthelmintic drugs. For a better understanding of the response of *H. contortus* to IVM and for the screening of drug-resistance-related genes, we used RNA sequencing and isobaric tags for relative and absolute quantification (iTRAQ) technology to detect the transcriptomic and proteomic changes in *H. contortus* after ivermectin treatment. An integrated analysis of the two omics showed that the differentially expressed genes and proteins were significantly enriched in the pathways of amino acid degradation, the metabolism of xenobiotics by cytochrome P450, the biosynthesis of amino acids, and the tricarboxylic acid cycle. We found that the upregulated UDP-glycosyltransferases (*UGT*), glutathione S-transferase (*GST*), cytochrome P450 (*CYP*), and p-glycoprotein (*Pgp*) genes play important roles in drug resistance in *H. contortus*. Our work will help in the understanding of the transcriptome and proteome changes in *H. contortus* after IVM and will facilitate the discovery of genes related to drug resistance. This information can be further applied to increase the understanding of the response of IVM in relation to *H. contortus*.

## 1. Introduction

*Haemonchus contortus* is one of the most pathogenic gastrointestinal nematodes infecting small ruminants worldwide. It feeds on the blood of ruminant abomasum, especially that of sheep and goats. Infection by this blood-sucking nematode causes the symptoms of haemonchosis, which include anemia, diarrhea, weight loss, and even death in cases of severe infection [1]. Furthermore, milk and meat production can be reduced in infected animals, causing tremendous economic losses to the agricultural industry worldwide. However, no vaccines protecting against these parasites are currently available; the primary form of nematode control is the use of anthelmintic drugs [2,3,4,5].

Ivermectin (IVM) is the first commercially available macrocyclic lactone endectocide. Since its introduction into the agricultural market in the early 1980s, it has been used to treat a wide variety of nematode parasites, including *H. contortus*, and it has quickly proven to be a very effective drug [6,7,8]. However, despite its benefits, the widespread usage of IVM has resulted in serious problems related to drug resistance worldwide, especially in countries where livestock husbandry is the dominant industry. In a vicious cycle of use and resistance, as IVM use increases, parasite resistance increases accordingly, which necessitates the use of yet more drugs [9,10,11]. Therefore, the problem of drug resistance has become an international issue that urgently requires a solution.

Drug resistance is the tolerance of a parasite to a given drug. This problem arose in conjunction with IVM five years after its initial use in the control of parasitic diseases [12]. It can therefore be inferred that the development of drug resistance is a gradual process. According to reports, IVM can be used as an agonist of glutamate to enhance the opening frequency of glutamate-gated chloride (GluCl). A low concentration of ivermectin can enhance the action of neurotransmitters, and a high concentration of ivermectin can enhance cell membrane permeability to chloride ions, leading to blocked nerve conduction and muscle relaxant paralysis. After the peristalsis of the pharyngeal muscles of *H. contortus* is blocked, the feeding of *H. contortus* is disturbed or hindered, which eventually leads to the starvation and death of *H. contortus* [13,14,15]. Many studies have shown that nematode ATP-binding cassette (ABC) transport proteins, including P-glycoproteins (Pgps), play an important role in anthelmintic resistance [16,17,18]. One study showed that Pgps act as efflux pumps to expel hydrophobic xenobiotics from cells [19,20]. According to the report, the ability of multidrug-resistant *Teladorsagia circumcincta* to survive IVM exposure may be associated with the increased expression of *Pgp-9* and gene sequence polymorphism [21]. Similarly, the expression levels of Pgps in *Caenorhabditis elegans* after exposure to IVM and the sensitivity of *Pgp* knock-out strains of *C. elegans* to IVM are both increased [2,22]. Furthermore, *Pgp-9.2* may be one of the most relevant candidates contributing to the multi-genic nature of the IVM resistance trait [23]. Cwiklinski et al. (2013) found that *glc-3* of *Cylicostephanus goldi* is one of the primary targets of macrocyclic lactone anthelmintics through a transcriptome analysis [24].

Anthelmintic resistance can be inherited, as its development requires the existence of resistance genes. The study of these drug-resistant genes is therefore the first step in understanding parasite resistance. Drug resistance is the result of the common regulation of multiple genes, rather than being attributable to a single gene [25,26,27]. Studying drug resistance is therefore vital for monitoring and controlling its further evolution and for delaying the accumulation of drug-resistance-related genes. However, the study of drug resistance is still in relatively early stages; drug-resistance-related genes are not comprehensively understood, and there are many such genes that need to be explored. It is urgent to identify genes that are potentially related to drug resistance. The objective of the present study was to use high-throughput sequencing combined with a bioinformatics analysis to investigate the changes in gene expression in *H. contortus* in both resistant and sensitive strains before and after IVM treatment. This study shows that IVM can cause transcriptional and proteomic changes in *H. contortus*.

## 2. Materials and Methods

### 2.1. Ethics Approval

The study design was reviewed and approved by the Animal Ethics Committee of Ningxia University (Permit No. 22-031). The procedures involving animals were carried out in accordance with the Animal Ethics Procedures and Guidelines of the People’s Republic of China. All efforts were made to minimize suffering and to reduce the number of sheep used in the experiment.

### 2.2. Sample Collection

The IVM-susceptible and -resistant *H. contortus* strains were isolated in our laboratory at Inner Mongolia Agricultural University, where they have been maintained for several years [28]. All experimental sheep were newborn lambs from our laboratory in Hohhot. Each animal was housed in a single pen and had free access to food and water. Fecal samples were collected and examined using the McMaster technique at regular intervals to ensure that the nematode egg counts of all sheep showed negative values (mean fecal egg count = 0 eggs per gram). After a week of feeding, the sheep were infected with approximately 10^4^ of either susceptible or resistant *H. contortus* in the L3 stage. The health of all infected sheep was monitored closely. After 20 days, feces were collected from each infected animal and placed into corresponding boxes with small holes, which were marked with the collection date and strain number. For the recovery of *H. contortus*, the boxes were incubated at 27 °C for approximately one week, and the fecal samples were slightly moistened with tap water as necessary (e.g., under dry conditions). After approximately one week, *H. contortus* at the L3 stage were collected with a self-made funnel separator, rinsed thoroughly with deionized water, and stored at 15 °C for further use.

### 2.3. Ivermectin Treatment

The two treatment groups were susceptible larvae treated with IVM (S1) and resistant larvae treated with IVM (R1). The two control groups, S0 and R0, were L3 larvae from susceptible and resistant strains, respectively, that had not been treated with IVM. The final concentration of IVM in the treatment groups (S1 and R1) was 0.28 μM, while the control groups (S0 and R0) were treated with a fresh medium without IVM. Each group included three biological replicates. All samples were cultured for 24 h and then harvested and stored in liquid nitrogen until used for RNA extraction, RNA-seq, and iTRAQ.

### 2.4. Transcriptomics

#### 2.4.1. RNA Extraction, Sequencing, and Identification of Differentially Expressed Transcripts (DETs)

Lysis Buffer (600 µL) was added to the sequencing sample, and then the total RNA was individually extracted from each sample using a mirVana miRNA Isolation Kit (Ambion, Shanghai, China) following the manufacturer’s protocol (Supplementary Materials Table S1). RNA integrity was evaluated using an Agilent 2100 Bioanalyzer (Agilent Technologies, Santa Clara, CA, USA). The samples with an RNA integrity number ≥ 7 were subjected to subsequent analyses. Libraries were constructed using a TruSeq Stranded mRNA LTSample Prep Kit (Illumina, San Diego, CA, USA) according to the manufacturer’s instructions. The libraries were sequenced, performed on an Illumina sequencing platform (HiSeqTM 2500, Illumina, San Diego, CA, USA), and 150 bp paired-end reads were generated.

#### 2.4.2. Sequence Filtering, Functional Annotation, and Analysis of Differentially Expressed Genes

Raw data were processed using Trimmomatic [29] to remove low-quality reads and those containing ploy-N in order to obtain clean reads. The clean reads were assembled into expressed sequence tag clusters (contigs) and assembled de novo into transcripts with Trinity [30] using the paired-end method. The longest transcript was chosen according to similarity for subsequent analyses. All downstream analyses were based on the clean, high-quality reads. Blastx [31] was used to annotate the unigenes by aligning these with the following NCBI databases: nonredundant protein (NR), SwissProt, and Clusters of Orthologous Groups (COG) for *C. elegans* complete genomes. In addition, the proteins with the highest number of unigene hits were assigned functional annotations. Based on the SwissProt annotation, Gene Ontology (GO) classification was performed by mapping the associations between the SwissProt sequences and the GO terms; to annotate potential metabolic pathways, the unigenes were mapped to the Kyoto Encyclopedia of Genes and Genomes (KEGG) database [32].

Fragments per kilobase of exon per million fragments mapped (FPKM) [33] and the read count values of each unigene were calculated using Bowtie 2 [34] and eXpress [35]. The differentially expressed genes (DEGs) of each group (three replicates per group) were identified using the DESeq [36] R package functions estimateSizeFactors and nbinomTest. A gene with a *p*-value < 0.05 and log2foldchange ≥ 1 or ≤−1 was considered to be differentially expressed. GO and KEGG pathway enrichment analyses of DEGs were performed using R based on the hypergeometric distribution. RNA-seq, read alignment, and DEG identification were carried out at OE Biotech (Shanghai, China).

### 2.5. Quantitative Proteomics (iTRAQ)

#### 2.5.1. Protein Extraction, Quantization, and SDS-PAGE Electrophoresis

The frozen samples were removed and ground thoroughly in the presence of liquid nitrogen. A mixture of phenol extraction solution and PMSF (600 µL) was added to attain a final concentration of 1 mM. The samples were further lysed via sonication (1 s ntervals, 3 min, 80 W). A phenol-Tris-HCl (pH 7.8) saturated solution was added and shaken (30 min, 4 °C). The mixtures were centrifuged (7100× *g*, 10 min, 4 °C) to collect phenol supernatants. The supernatants were added to five volumes of 0.1 M cold ammonium acetate–methanol buffer and precipitated at −20 °C overnight. The precipitate was washed with five volumes of cold methanol and centrifuged again (12,000× *g*, 10 min, 4 °C) to remove more precipitate. This process was then repeated. Methanol was replaced with acetone, and the wash step was performed twice. The samples were centrifuged (12,000× *g*, 10 min, 4 °C) to collect the precipitate, which was dried at room temperature and dissolved in lysis buffer for 3 h. The samples were centrifuged, and the supernatants were collected. The supernatants were centrifuged again to remove precipitates completely. Protein concentration was determined using the BCA method [37], and the proteins were then stored at −80 °C. Additionally, 7 μg samples were subjected to 12% SDS-PAGE, visualized, and scanned according to Candiano’s protocol [38].

#### 2.5.2. Proteolysis and ITRAQ Labeling

The FASP method [39] was adopted for the enzymatic hydrolysis of the proteins (100 μg), and the labeling peptide solutions were lyophilized and stored at −80 °C.

#### 2.5.3. Reversed-Phase Liquid Chromatography (RPLC)

Reversed-phase liquid chromatography was performed on an 1100 HPLC System (Agilent) using an Agilent Zorbax Extend RP column (5 μm, 150 mm × 2.1 mm). The elution buffer was collected every 1 min and placed in turn into a 1–15 centrifuge tube (Thermo Fisher Scientific, Waltham, MA, USA); samples were harvested from 8 min to 60 min. After collection, the samples were vacuum freeze-dried and cryopreserved for MS detection.

#### 2.5.4. LC-MS/MS Analysis and Data Processing

The samples were loaded using a capillary C18 trap column (3 cm × 100 µm) and separated using a C18 column (15 cm × 75 µm) on an Eksigent nanoLC-1D Plus System (SCIEX, Framingham, MA, USA). An analysis was performed using a TripleTOF 5600 mass spectrometer (SCIEX, Framingham, MA, USA) equipped with a Nanospray III source (SCIEX, Framingham, MA, USA). All raw LC-MS/MS data were searched against the sample protein database using Proteome DiscovererTM 2.2 software (Thermo, USA). At least two peptides are required for a peptide group to be considered for the purpose of quantification; the false positive rate of peptide identification was controlled below 1%.

### 2.6. Validation of RNA-Seq Results Using Quantitative Real-Time PCR (q-PCR)

The expressions of DEGs in different groups were detected using quantitative real-time PCR (q-PCR) to confirm the RNA-seq-based transcriptional response of the susceptible and resistant strains of L3-stage *H. contortus* before and after IVM treatment. Genes that were upregulated or downregulated were identified by performing a sequencing analysis. The RNA samples were reverse-transcribed to single-stranded cDNA using a PrimeScript^TM^ RT Reagent Kit (TaKaRa, Dalian, China). The same samples were used for sequencing and q-PCR. β-tublin was used as the reference gene, and the DEGs used for q-PCR verification were randomly selected. TB Green^®^ Premix Ex Taq™ II (TaKaRa, Dalian, China) was used to perform q-PCR according to the manufacturer’s instructions. The selected genes were analyzed in triplicate; the forward (F) and reverse (R) primers used in q-PCR are listed in Table S2. The q-PCR cycling was performed under the following conditions: 95 °C for 30 s, followed by 40 cycles of 95 °C for 5 s, 55 °C for 30 s, 95 °C for 10 s, and a melting curve analysis ranging from 65 °C to 95 °C. The 2^−ΔΔCT^ relative expression method was used to calculate the expression of each gene.

## 3. Results

We used the Illumina HiSeqTM 2500 platform with the cDNA libraries from IVM-treated *H. contortus* and obtained over 48,000,000 raw reads from each sample and more than 47,000,000 clean reads after processing (Supplementary Materials Table S3). A total of 69,728 unigenes were spliced, with a total length of 62,243,154 bp and an average length of 892 bp. The correlation coefficient of the unigene expression level among the different samples was close to 1, which indicates a high similarity of expression patterns between the samples (Supplementary Materials Figure S1). The sequencing data determined in this work have been deposited in the National Center for Biotechnology Information (NCBI) Sequence Read Archive (SRA) database (https://www.ncbi.nlm.nih.gov/sra, accessed on 3 April 2022) under accession no. PRJNA663203.

### 3.1. Differentially Expressed Genes (DEGs) and Differentially Expressed Proteins (DEPs)

**Figure 1:**
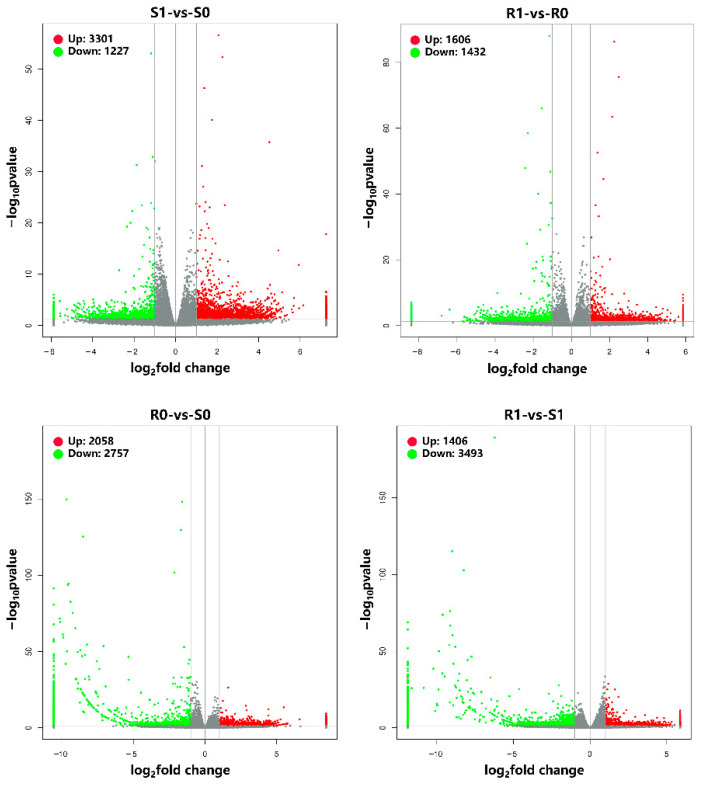
Volcano plots showing the transcriptional response of the four comparison groups. Differentially expressed genes (DEGs) are shown as red (upregulated) and green (downregulated) dots. Non-significantly expressed genes are shown as gray dots. The *X*-axis represents the value of log_2_ (fold change), and the *Y*-axis shows the value of −log_10_ (*p*-value).

**Figure 2:**
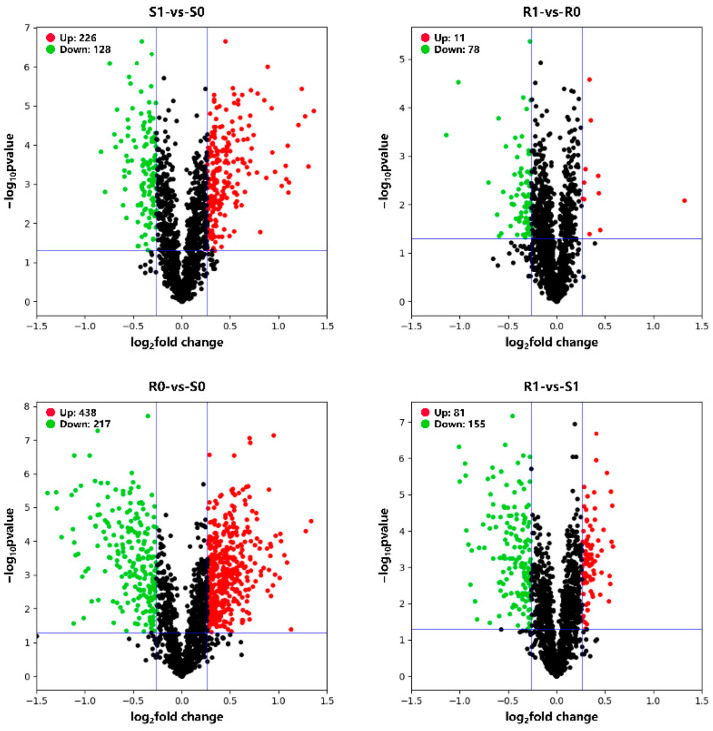
Volcano plots showing the differentially expressed proteins (DEPs) of the four comparison groups. DEPs are shown as red (upregulated) and green (downregulated) dots. Non-significantly expressed proteins are shown as black dots. The *X*-axis represents the value of log_2_ (fold change), and the *Y*-axis shows the value of −log_10_ (*p*-value).

**Figure 3:**
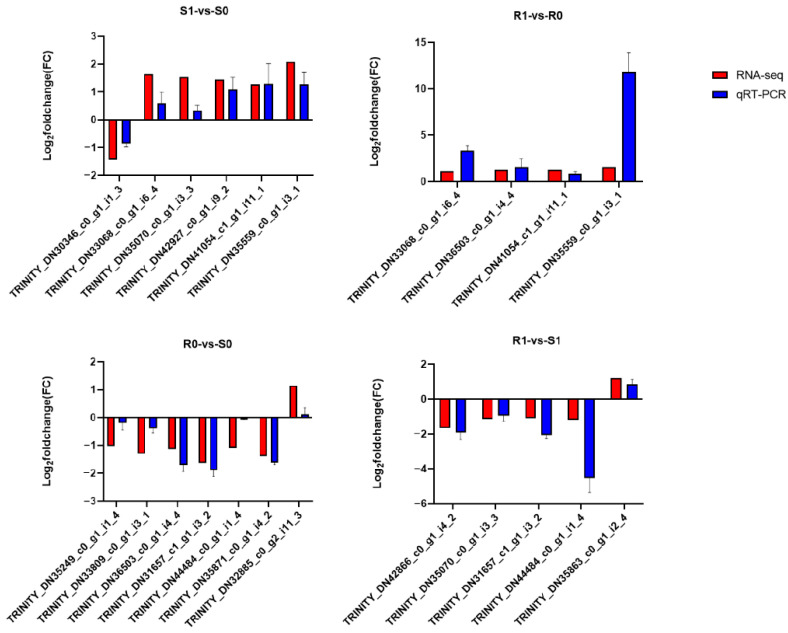
Validation of RNA-seq results using quantitative real-time PCR (qRT-PCR). The *X*-axis shows the genes that we tested, and the *Y*-axis represents the relative expressions of those genes.

The transcriptome analysis detected 3301 upregulated and 1227 downregulated genes in the susceptible *H. contortus* strain after IVM treatment (S0-vs-S1), while 1606 upregulated and 1432 downregulated genes were detected after IVM treatment in the resistant strain (R0-vs-R1). Additionally, 2058 upregulated and 2757 downregulated genes were detected in the non-treated resistant strain compared with the non-treated susceptible strain (S0-vs-R0), while 1406 upregulated and 3493 downregulated genes were detected in the IVM-treated resistant strain compared with the IVM-treated susceptible strain (S1-vs-R1; Figure 1). Regarding proteomics, a total of 1549 proteins were identified, of which 354 (226 upregulated and 128 downregulated), 89 (11 upregulated and 78 downregulated), 655 (438 upregulated and 217 downregulated), and 236 (81 upregulated and 155 downregulated) proteins were differentially expressed in the four comparison groups, (S0-vs-S1, R0-vs-R1, S0-vs-R0, and S1-vs-R1, respectively; Figure 2). We also found that the total number of DEPs was much lower than the total number of DEGs. The expressions of the genes obtained through the RNA-seq were confirmed using qPCR, and the validation results are shown in Figure 3.

### 3.2. Integrated Analysis of Transcriptome and Proteome

**Figure 4:**
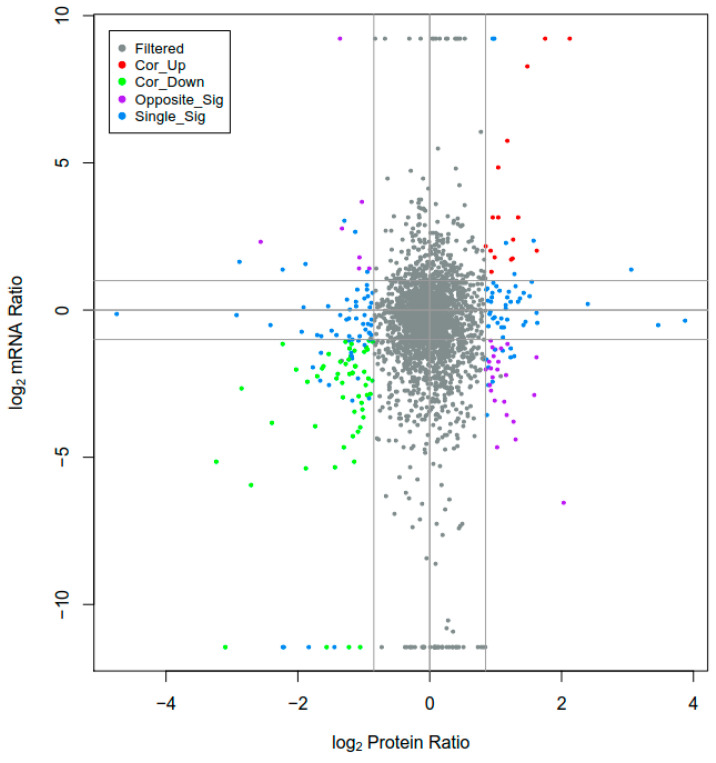
Relationship patterns of total mRNA and protein. The *X*-axis shows protein expression, and the *Y*-axis represents gene expression. Gray dots indicate genes and proteins with no significant difference, red dots represent upregulated genes and proteins, green dots represent downregulated genes and proteins, purple dots represent differentially expressed genes (DEGs) and differentially expressed proteins (DEPs) that show opposite regulation, and blue dots represent that one of the genes and proteins differ.

To identify the genes and proteins associated with drug resistance, we integrated the differentially expressed transcripts and proteins. As shown in Figure 4, almost all of the log_2_ mRNA:log_2_ protein ratios are concentrated in the center of the plot; these genes and proteins were filtered out, while the DEPs and DEGs were left in place. An integrated analysis of the transcriptome and proteome data revealed that the expressions of one gene and the corresponding protein (TRINITY_DN33068_c0_g1_i6_4) were upregulated and that two other genes (TRINITY_DN29476_c0_g1_i7_3 and TRINITY_DN42604_c4_g1_i6_1) were downregulated in the S0-vs-S1 group; one gene and the corresponding protein (TRINITY_DN35814_c0_g1_i8_2) were downregulated in the R1-vs-R0 group; one gene and the corresponding protein (TRINITY_DN43412_c0_g1_i1_3) were upregulated and nine were downregulated in the R0-vs-S0 group; and three genes and the corresponding proteins (TRINITY_DN30909_c0_g1_i3_1, TRINITY_DN38401_c0_g1_i1_1, and TRINITY_DN3919_c0_g1_i1_1) were downregulated in the R1-vs-S1 group (Supplementary Materials Figure S2).

### 3.3. Gene Ontology (GO) Analysis of DEGs and DEPs

**Figure 5:**
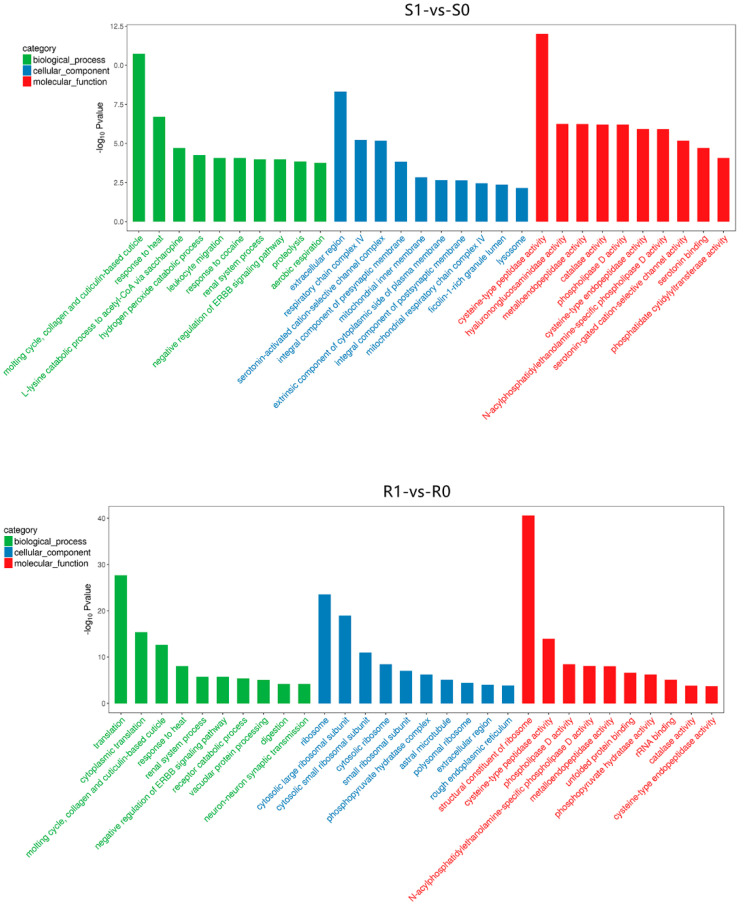

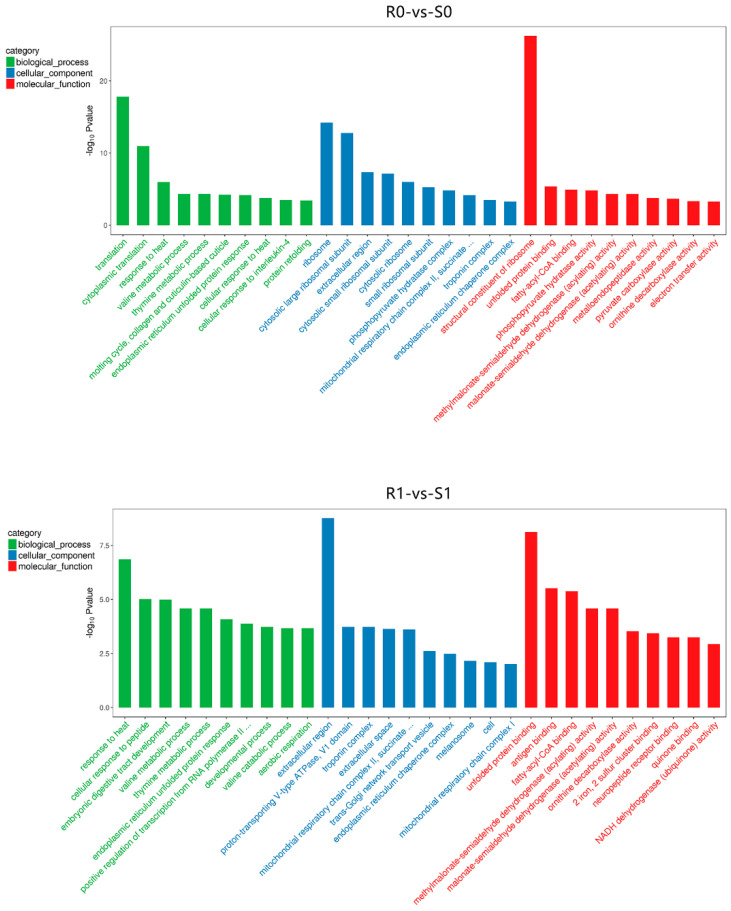
The 30 most significantly enriched gene ontology (GO) terms for the DEGs in the four comparison groups. The GO analysis results are summarized in three categories: biological processes (green), cellular components (blue), and molecular functions (red). The *X*-axis indicates different GO terms, and the *Y*-axis represents the corresponding number of genes in each GO term.

**Figure 6:**
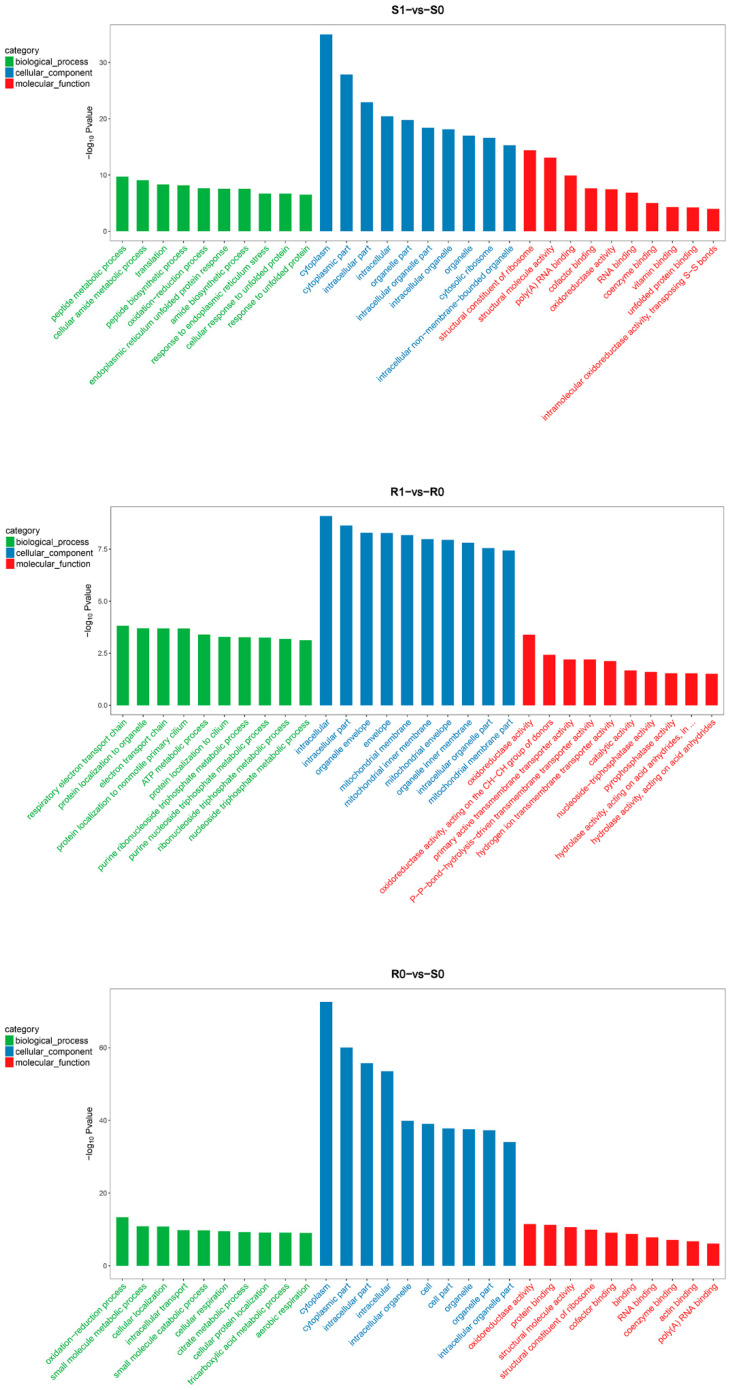

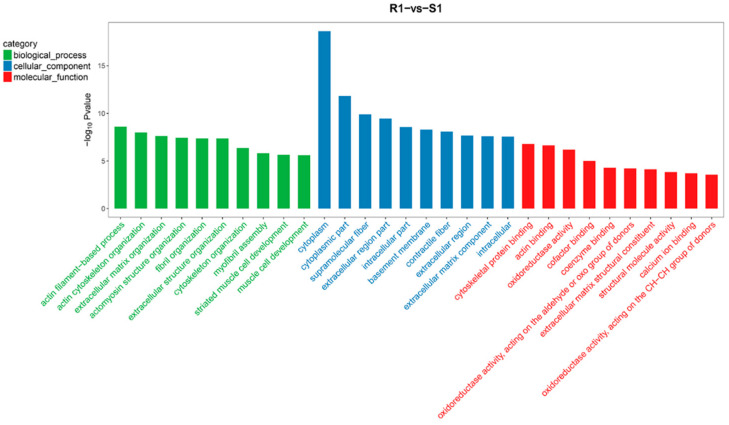
The 30 most significantly enriched gene ontology (GO) terms for the DEPs in the four comparison groups. The GO analysis results are summarized in three categories: biological processes (green), cellular components (blue), and molecular functions (red). The *X*-axis indicates different GO terms, and the *Y*-axis represents the corresponding number of genes in each GO term.

The GO enrichment analysis showed dynamic differences in the biological processes, cellular components, and molecular functions of *H. contortus* before and after IVM treatment. Based on the −log_10_ *p*-value, we list the top 30 GO terms for DEGs and DEPs of different comparison groups (Figure 5 and Figure 6). Most of the GO terms in the biological process category for DEGs were associated with metabolic processes and catalytic activity, while those for DEPs were mainly involved in redox and catabolism.

In the cellular component category, DEGs showed significant enrichment in the extracellular region (GO:0005576), ribosome (GO:0005840), and cytosolic large ribosomal subunit (GO:0022625), while DEPs were mainly classified in the intracellular organelle region (GO:0044446), cytoplasm (GO:0005737), and mitochondrial membrane (GO:0044455). In the molecular function category, DEGs were enriched in metalloendopeptidase activity (GO:0004222), structural constituents of ribosomes (GO:0003735), and cysteine-type peptidase activity (GO:0008234; Figure 5), while enriched DEPs were mainly related to oxidoreductase activity (GO:0016635), structural molecule activity (GO:0005198), and structural constituents of ribosomes (GO:0003735; Figure 6). The other enriched terms shared by DEGs and DEPs (not shown in the figure) were response to drug (GO:0042493), drug transmembrane transport (GO:0006855), negative regulation of response to drug (GO:2001024), drug binding (GO:0008144), and drug transmembrane transporter activity (GO:0015238). In summary, the GO enrichment analysis further showed that the orderly cooperation of biosynthesis, decomposition, metabolism, and transmembrane transport collectively maintained the metabolism and homeostasis of *H. contortus*.

### 3.4. KEGG Pathway Analysis of the DEGs and DEPs

**Figure 7:**
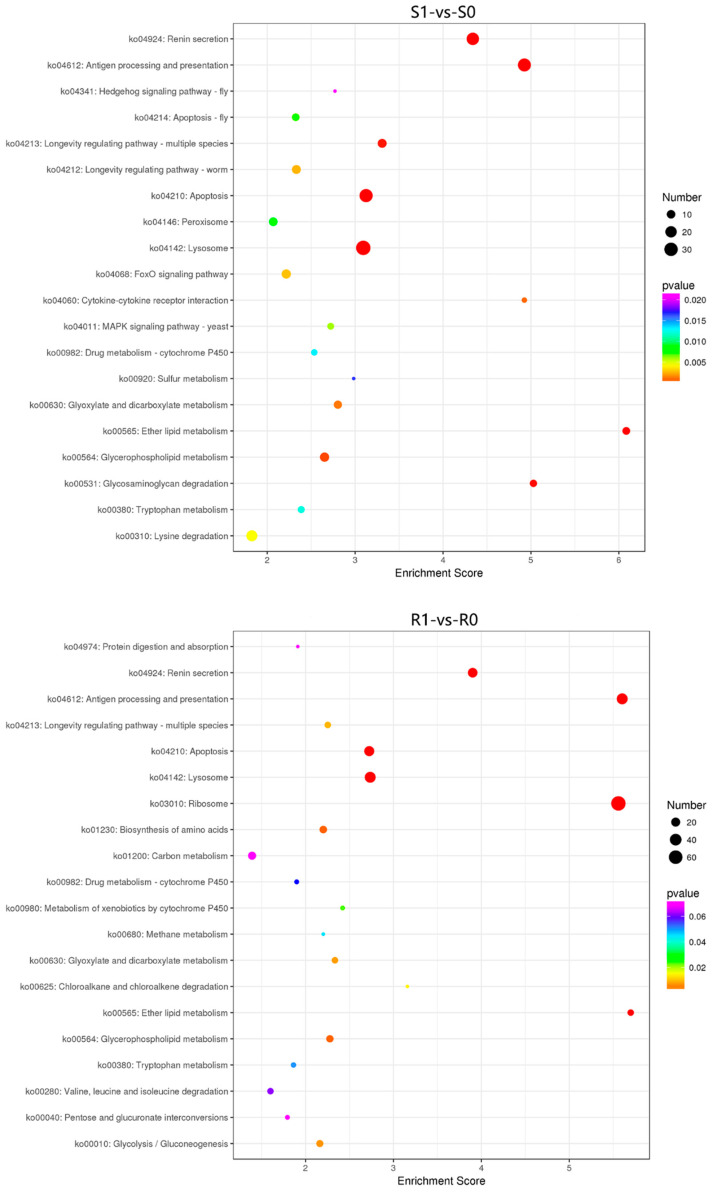

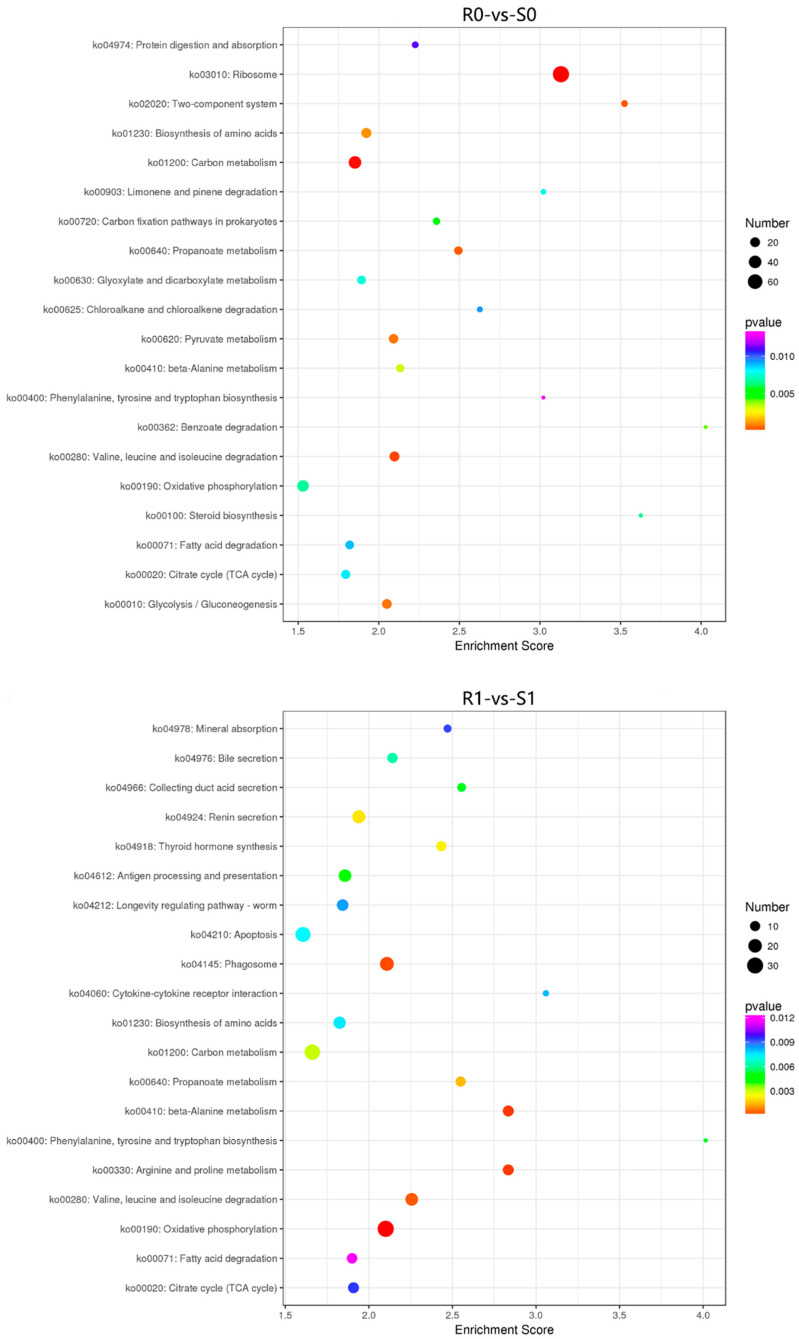
Scatterplot of the top 20 most enriched KEGG enrichment pathways for the DEGs in the four comparison groups. The *X*-axis indicates enrichment score, and the *Y*-axis represents the distinct KEGG pathways.

**Figure 8:**
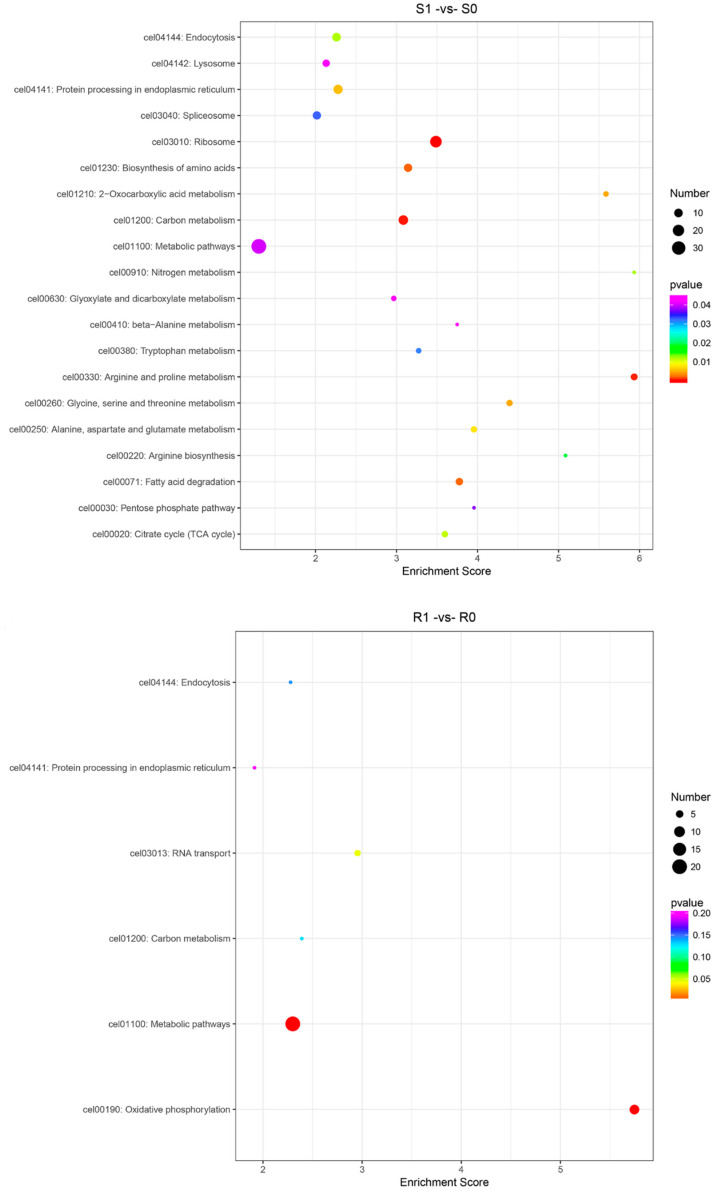

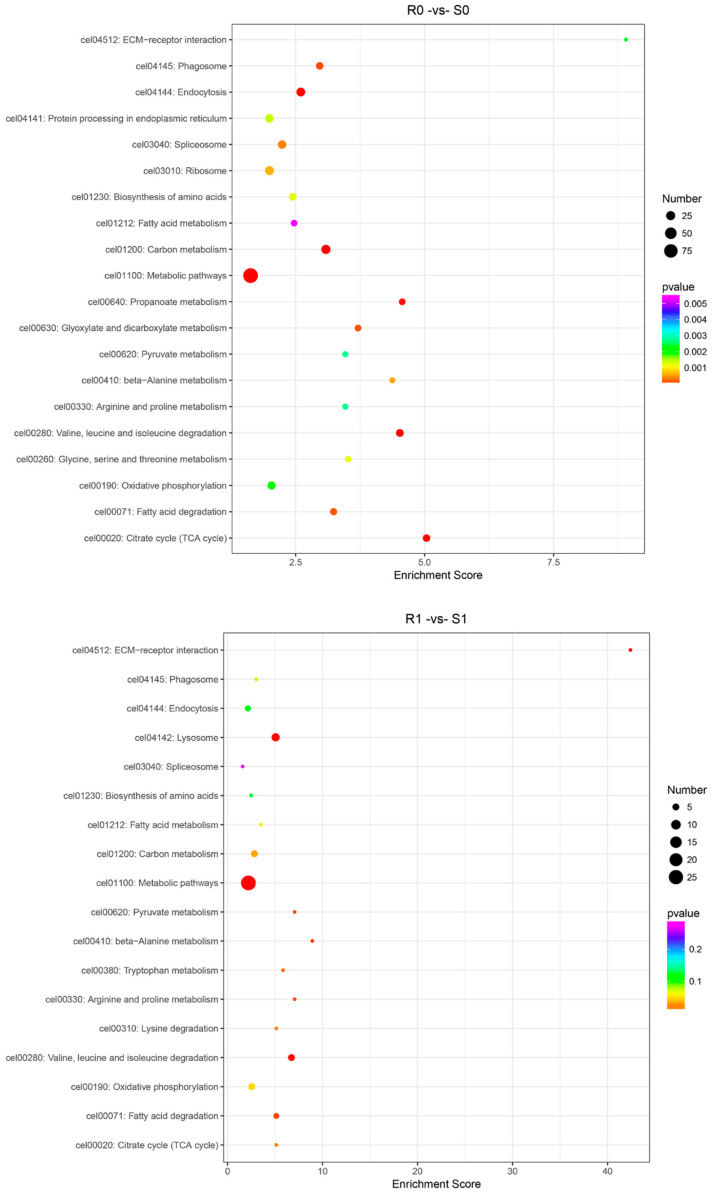
Scatterplot of the top 20 most enriched KEGG enrichment pathways for the DEPs in the four comparison groups. The *X*-axis indicates enrichment score, and the *Y*-axis represents the distinct KEGG pathways.

The KEGG enrichment analysis of the DEGs and DEPs revealed 4528 DEGs in the 186 KEGG pathways of the S1-vs-S0 group; 3038 DEGs were enriched in the 170 KEGG pathways of the R1-vs-R0 group; 4815 DEGs were enriched in the 231 KEGG pathways of the R1-vs-R0 group; and 4899 DEGs were enriched in the 230 KEGG pathways of the R1-vs-R0 group. We selected the 20 most significantly enriched KEGG pathways according to the enrichment scores. These DEGs were significantly enriched in the pathways related to xenobiotic metabolism by cytochrome P450, amino acid degradation, the biosynthesis of amino acids, drug metabolism–cytochrome P450, carbon metabolism, and the tricarboxylic acid (TCA) cycle (Figure 7). The DEPs were significantly enriched in the pathways related to carbon metabolism, the TCA cycle, endocytosis, ABC transporters (MRPs, Pgps), and drug metabolism–other enzymes (UGT, GST) (Figure 8). Overall, these DEGs and DEPs were enriched in pathways related to the decomposition, metabolism, and synthesis of substances; this result is consistent with that of the GO enrichment analysis. These genes and proteins may play an important role in the response to the metabolism of anthelmintics in susceptible and resistant strains.

## 4. Discussion

Drug resistance is usually defined as the ability of an organism to survive a given dose of drugs. Partly because of its large impact on economic development in most parts of the world, *H. contortus* is the most widely studied nematode in terms of drug resistance. The focus on this topic in this species has led to an upsurge in research into drug resistance. At the same time, due to its biological and physiological factors, such as its high fecundity, relatively large body size, and simple larval storage conditions, *H. contortus* is regarded as a good experimental model [40,41,42]. Omics has become an important research tool for exploring the molecular mechanisms related to drug resistance and for identifying genes related to drug resistance [43]. We therefore used omics sequencing techniques to analyze and investigate the expressions of different genes and proteins in resistant and sensitive strains of *H. contortus*.

In this study, transcriptomic and proteomic sequencing techniques were used to evaluate the global transcriptomic and proteomic changes in *H. contortus* after IVM treatment. We found that 4528 genes in susceptible strains and 3038 genes in resistant strains were significantly regulated after IVM treatment (Figure 1), which indicates that IVM had significant effects on the gene and protein expressions in *H. contortus*, especially in susceptible strains. Conversely, 354 and 89 proteins were significantly regulated in the susceptible and resistant strains, respectively, after IVM treatment. The apparent quantitative contrast between the genes and proteins reflects the proteome–transcriptome complexity, and the result of this comparison may be due to the fact that most of the genes encoded either hypothetical or non-functional annotated proteins. Thus, there is still a large knowledge gap in our understanding of transcriptional responses under IVM treatment. GO and KEGG pathway enrichment analyses are downstream procedures that are commonly used to interpret differential expression results [44]. Some of the genes we were interested in were upregulated or downregulated; we used annotation as part of our GO and KEGG analyses in order to determine which terms or pathways were significantly enriched [45]. The research objectives of the two omics are the same, and there must be a certain correlation between the groups. The transcriptome and proteome association analyses showed that the genes and proteins that are associated with GTP, RNA, proteolysis, synthesis, and catabolism terms were upregulated under IVM treatment, indicating that the energy and protein production rates were increased in *H. contortus* after IVM treatment. It has been reported that the genes involved in these functions are also upregulated in *H. contortus* after albendazole (ABZ) treatment [46] and in *Acinetobacter baumannii* after antibiotic treatment [47]. This phenomenon may be caused by the drug delivery screening that the organisms are subjected to in determining resistant strains, with selective pressure leading to the upregulation of resistance-related gene expression in the presence of the drug. In addition, we found that a single drug-resistant gene did not necessarily lead to IVM resistance in *H. contortus*. An extensive network of resistance-related genes, such as MRPs and Pgps, play a protective role in response to the efflux of anthelmintics in susceptible and resistant strains. In this study, these genes and proteins were significantly regulated in both susceptible and resistant strains.

The transcriptome and proteome KEGG enrichment analyses showed that some pathways associated with drug metabolism, such as that of xenobiotics by cytochrome P450 (cel00980) and drug metabolism–cytochrome P450 (cel00982), were activated after IVM treatment. Cytochrome P450 (*CYP*) is involved in a variety of biosynthetic, catabolic, and xenobiotic detoxification functions [48]. The relationship between *CYP* expression and drug resistance has been demonstrated in insects. It has been reported that multi-insecticide resistance in *Drosophila simulans* is associated with the overexpression of *CYP6g1* [49,50] and that pyrethroid resistance is associated with the overexpression of *CYP6P9* in *Anopheles funestus* [51]. Additionally, some studies suggest the opposite. For example, some proteins of *C. elegans*, especially members of the *CYP35* family, have been shown to be inducible by exogenous organisms [52,53]. ABZ can induce the expression of multiple *CYP* genes in *C. elegans* [54], while the inhibitor of *CYP*, piperonyl butoxide (PBO), increases the toxicity of the insecticide rotenone to *H. contortus* larvae and adults [55]. In this study, a comparative analysis showed that the CYPs of *H. contortus* were significantly regulated after IVM treatment.

Differences at the molecular level in susceptible strains before and after IVM treatment play a role in the study of drug resistance. The transcriptome and proteome association analysis of the expressions of differential genes and proteins in the S1-vs-S0 group revealed that the expression of only one gene, glutathione S-transferase (*GST*; TRINITY_DN33068_c0_g1_i6_4), in the IVM-treated susceptible strains showed the same trend (upregulation) as the corresponding protein (Supplementary Materials Figure S2). This gene was enriched in the pathways of xenobiotic metabolism by cytochrome P450 (cel00980), drug metabolism–cytochrome P450 (cel00982), and glutathione metabolism (cel00480). It has been reported that the activity of glutathione S-transferase (*GST*) is 1.5–1.8 times higher in the cambendazole-resistant strains of *H. contortus* than in susceptible strains [56]. The activity and expression of *GST* genes are upregulated in a dose-dependent manner in *Helicoverpa armigera* larvae after pesticide exposure [57]. Pugazhendhi et al. (2017) found that the antibiotic-induced *GST* activity of bacteria from a poultry litter was 3–4 times higher than that of the control, which led to the speculation that *GST* plays an important role in antibiotic resistance [58]. In addition, *GST* is involved in drug resistance in organisms such as *Musca domestica*, *Bombyx mori*, and *Aedes aegypti* [59,60,61,62]. In this study, the expression of *GST* in the drug-resistant strains was three times higher than that in the susceptible strains. This gene upregulation may enable resistance to the pressure of drug selection before the susceptible strain becomes resistant to the drug. In addition, we found that the *UGT* (TRINITY_DN44820_c0_g1_i7_2) gene, which is related to detoxification in organisms, was significantly enriched in transcriptome and proteome association pathways. According to one study, the UDP-glycosyltransferases (*UGT*) inhibitor chrysin reduces ABZ biotransformation in *C. elegans* [63]. Matoušková et al. (2018) found that the expression of *UGT* in drug-resistant strains of *H. contortus* was significantly higher than that in susceptible strains [64].

In this study, the expression of *UGT* was upregulated in drug-resistant strains before and after treatment and in both strains after IVM treatment; the expression was higher in resistant strains than in susceptible strains. The significant differences in the transcription and protein levels support the possibility of important roles for *UGT* and *GST* in general drug resistance; however, the role played by this upregulated transcription is currently unknown. Additionally, it remains to be elucidated whether the *UGT* and *GST* genes are involved in the biotransformation of IVM anthelmintics and how significant a role they play in resistance. The genes and pathways that we identified here may serve as therapeutic targets to control the further development of drug resistance. Further research will provide insights into the function of the various DEGs, elucidating the mechanisms of drug resistance, thereby potentially enabling these to be overcome through targeted therapy.

## 5. Conclusions

In this study, we identified and evaluated the genes and pathways related to IVM resistance in *H. contortus* using an integrated transcriptomic and proteomic analysis. We found that 1432 and 1227 genes were downregulated in the IVM-treated resistant and susceptible strains, respectively, suggesting that IVM inhibits the expression of some of the genes in *H. contortus*. Among the many upregulated genes that we uncovered, we focused on the changes in *UGT*, *GST*, and the *CYP* genes. These DEGs and their associated DEPs were significantly enriched in RNA, proteolytic synthesis, catabolic functions, and some metabolism-related pathways. To sum up, our study provides useful information for a better understanding of the response of *H. contortus* to IVM and for the screening of genes that may be associated with drug resistance.

## Data Availability

Not applicable.

## Supplementary Materials

The following supporting information can be downloaded at: https://www.mdpi.com/article/10.3390/ani13050919/s1, Figure S1: A heatmap showing the magnitude of the Pearson’s correlation coefficient matrix among different groups, Figure S2: Venn diagram showing the co-upregulated and co-downregulated DEGs and DEPs in four groups, Table S1: RNA extraction procedure, Table S2: Primer sequences used for q-PCR analysis, Table S3: Quality metrics of the clean reads.
