# Supplementary material for: Transcriptomics and Proteomics of Haemonchus contortus in Response to Ivermectin Treatment
Source: Animals (Basel). 2023 Mar 3;13(5):919. doi: 10.3390/ani13050919 (PMC10000067; doi:10.3390/ani13050919)
Supplement: Supplementary file 1 [file animals-13-00919-s001.zip › Supplementary Materials Table S1.pdf]

RNA Extraction

**Step 1.** Add 600 μL Lysis/Binding Buffer to the cell or tissue lysate. Then add 30 μL miRNA Homogenate Additive to homogenate, and mix well by vortexing or inverting the tube several times. Leave the mixture on ice for 10 min.
**Step 2.** Add a volume of Acid-Phenol: Chloroform that is equal to the lysate volume before addition of the miRNA Homogenate Additive. Centrifuge for 5 min at maximum speed (10,000 x g) to separate the aqueous and organic phases. Carefully remove the aqueous (upper) phase without disturbing the lower phase, and transfer it to a fresh Tube.
**Step 3.** Add 1.25 volumes of room temperature 100% ethanol to the aqueous phase.
**Step 4.** Pipet the lysate/ethanol mixture (from the previous step) onto the Filter Cartridge (Note: Up to 700 μL can be applied to a Filter Cartridge at a time.). Centrifuge for 30 sec at 13,000 rpm to pass the mixture through the filter. Discard the flow-through.
**Step 5.** Apply 350 μL miRNA Wash Solution 1 to the Filter Cartridge and centrifuge for 30 sec at 13,000 rpm. Discard the flow-through from the Collection Tube, and replace the Filter Cartridge into the same Collection Tube.
**Step 6.** The 10 μL DNase I and 70 μL Buffer RDD QIAGEN (#79254) were mixed. Then the mixture was added to the Filter Cartridge. Leave it at the room temperature for 15 min.
**Step 7.** Apply 350 μL miRNA Wash Solution 1 to the Filter Cartridge and centrifuge for 30 sec at 13,000 rpm. Discard the flow-through from the Collection Tube, and replace

the Filter Cartridge into the same Collection Tube.
**Step 8.** Apply 500 µL Wash Solution 2/3 and centrifuge for 30 sec at 13,000 rpm. Draw it through the Filter Cartridge as in the previous step. Repeat with a second 500 µL aliquot of Wash Solution 2/3.
**Step 9.** Spin the assembly for 1 min to remove residual fluid from the filter. Transfer the Filter Cartridge into a fresh Collection Tube. Apply 100 µL of pre-heated (95°C) Elution Solution to the center of the filter. Leave it at the room temperature for 2 min. Spin for ~20–30 sec at maximum speed to recover the RNA. Collect the eluate (which contains the RNA) and store it at –70°C.
