# Supplementary material for: Transcriptomics and Proteomics of Haemonchus contortus in Response to Ivermectin Treatment
Source: Animals (Basel). 2023 Mar 3;13(5):919. doi: 10.3390/ani13050919 (PMC10000067; doi:10.3390/ani13050919)
Supplement: Supplementary file 1 [file animals-13-00919-s001.zip › Supplementary Materials Table S2.pdf]

Additional File 1: Table S2. Primer sequences used for qPCR analysis.

Gene ID	Forward primer	Reverse primer
TRINITY_DN40923_c0_g1_i6_2	GCCCACCACAAGAGTGCCATTG	TGTTGCGTGCGATGCGTACTG
TRINITY_DN42866_c0_g1_i4_2	ACCAGGTCTACCACCAGGATGG	TGTCGTCGTGCCCTTGAATTGC
TRINITY_DN35249_c0_g1_i1_4	CGCCGTCACAGGTCCTGAGG	AGCCGTCCATCGTCAGCAAATG
TRINITY_DN30346_c0_g1_i1_3	CCACCCGAAGCCAGACACAATG	TGCAGCGGCCACGGAAAATAAG
TRINITY_DN33809_c0_g1_i3_1	AGTCGCATCGCAATGGGTCAAC	TGGCTCAGAGGTCGTCGTAACG
TRINITY_DN33068_c0_g1_i6_4	GCCTGGGAAGAAGCCGTTGTC	TTCTGGCGTGCAGGCTCAAAC
TRINITY_DN35070_c0_g1_i3_3	ACATCCACCCTGCCACTACCTC	GCCAAGAGCCAACAGAGATCCG
TRINITY_DN36503_c0_g1_i4_4	TCCTCGTTCTTCGGAGCCACTG	TGCCTGCACCGCCTCCTG
TRINITY_DN42927_c0_g1_i9_2	TCGGCTAGGCTCCTGCATCTC	CTTCCGCCCAACTTCCACTTCC
TRINITY_DN35863_c0_g1_i2_4	CAGTCAGCAGTCTCGGCTTTGG	ACCCACACGTAGACCCACATCC
TRINITY_DN41054_c1_g1_i11_1	CGCTGGTCAATGTCCGAGATGC	CGCACGGCTCATCACACTCTG
TRINITY_DN31657_c1_g1_i3_2	CACCTTCTCGGCTGATGCAGAG	ACAGACTCGCACCACTCACAGG
TRINITY_DN44484_c0_g1_i1_4	AACGCCCACACCACAATCACTG	TCCAGAAGCGGTCGGTGAGC
TRINITY_DN35559_c0_g1_i3_1	TGAAGCACAGCAGCAGACAAGC	TGACGAGTCCCTGGCAAGAACC
TRINITY_DN32885_c0_g2_i11_3	CCTGCTCGATCACGGCCTTTAC	CTCAGCCGCCACAGTTCTAAGC
TRINITY_DN35871_c0_g1_i4_2	GCTCTCACCACCAGCTCAAACC	GTTTGCGTCCAGAGAGGGCTTC
TRINITY_DN39784_c0_g1_i12_2	TTCCTGGCACCAAGCATGTCAC	AGAGGCGGTTACGAGGCTTCC
β-tublin	TGCTATGTTCCGTGGTCGTATG	CGGCAGTCTTAACGTTGTTTGG
