# Supplementary material for: Transcriptomics and Proteomics of Haemonchus contortus in Response to Ivermectin Treatment
Source: Animals (Basel). 2023 Mar 3;13(5):919. doi: 10.3390/ani13050919 (PMC10000067; doi:10.3390/ani13050919)
Supplement: Supplementary file 1 [file animals-13-00919-s001.zip › Supplementary MaterialsTable S3.pdf]

Additional File2: Table S3. Quality metrics of the clean reads

Sample	raw_reads	raw_bases	clean_reads	clean_bases	valid_bases	Q30	GC
R0_1	49264050	7389607500	48031754	6898484167	93.35%	94.83%	46.96%
R0_2	49312626	7396893900	48008416	6924796558	93.62%	94.76%	46.86%
R0_3	49474092	7421113800	48298698	7000416046	94.33%	94.84%	47.03%
R1_1	49146128	7371919200	48102856	6932801591	94.04%	95.18%	46.89%
R1_2	49485514	7422827100	48272780	6968416693	93.88%	94.61%	47.00%
R1_3	49372184	7405827600	48226140	6961794348	94.00%	94.82%	46.90%
S0_1	49606180	7440927000	48437128	6954565195	93.46%	94.80%	46.88%
S0_2	49298472	7394770800	48239314	6980366586	94.40%	95.03%	46.87%
S0_3	48945532	7341829800	47767524	6893094153	93.89%	94.44%	46.92%
S1_1	49775348	7466302200	48617334	7027730293	94.13%	94.81%	46.93%
S1_2	49620380	7443057000	48664172	7028595624	94.43%	95.22%	46.79%
S1_3	49721276	7458191400	48642132	7050589513	94.53%	94.95%	46.77%

Samples: Sample group names

raw_reads: Original reads number

raw_bases: Original sequencing volume

clean_reads: The reads number after filtering

clean_bases: The bases number after filtering

valid_base: Percentage of available bases

Q30: The percentage of bases whose Phred number is greater than 30 in Raw Bases

GC: The percentage of the total number of G and C in clean bases to the total number of bases
