# Supplementary figures and images for: Transcriptomics and Proteomics of Haemonchus contortus in Response to Ivermectin Treatment
Source: Animals (Basel). 2023 Mar 3;13(5):919. doi: 10.3390/ani13050919 (PMC10000067; doi:10.3390/ani13050919)

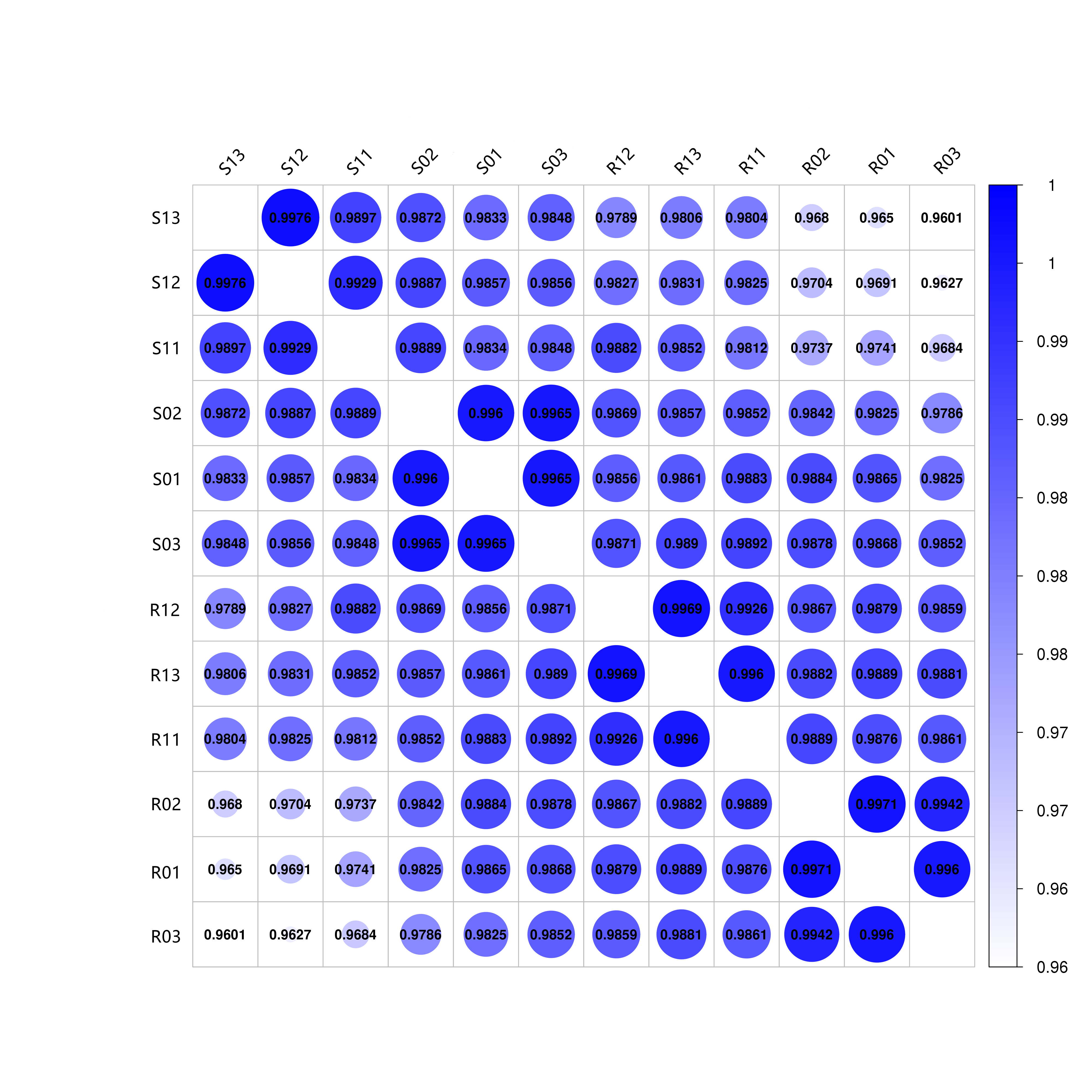

Supplement: Supplementary file 1 [file animals-13-00919-s001.zip › Supplementary Materials Figure S1.png]

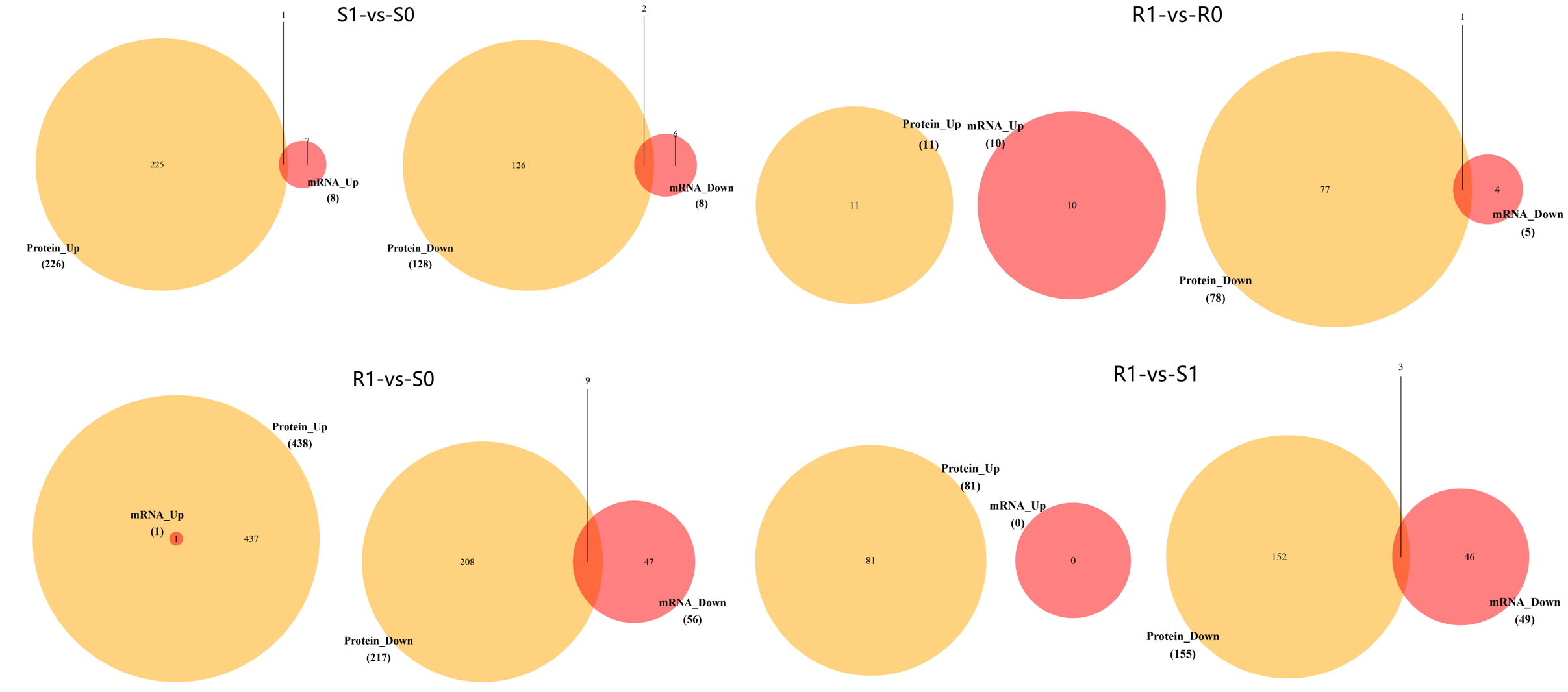

Supplement: Supplementary file 1 [file animals-13-00919-s001.zip › Supplementary Materials Figure S2.tif]
